# Empathy and Reversed Empathy of Stress in Mice

**DOI:** 10.1371/journal.pone.0023357

**Published:** 2011-08-10

**Authors:** Shigeru Watanabe

**Affiliations:** Department of Psychology, Keio University, Tokyo, Japan; Université Pierre et Marie Curie, France

## Abstract

Empathy is an emotional response to display of distress in others and reversed-empathy is an emotional response to non-distressed others in distressed subjects. Stress has memory enhancing effect on aversive experience. Here, I examine empathy and reversed empathy using the memory enhancing effects of stress in mice. Restrain stress enhanced aversive memory of a floor with electric shock, but restrain stress, with cage mates also restrained, reduced the enhancing effect. On the other hand, restrain stress with free-moving cage mates increased the memory enhancing effect, suggesting the stronger stress. This is the reversed-empathy. Level of corticosterone is the highest after the restrain with free-moving mates and lowest after the restrain with restrained mates.

## Introduction

Darwin believed that infrahuman animals have sense of empathy and wrote “many animals certainly sympathize with each other's distress or danger” [1]. Empathy is emotional response to display of distress in others. Exposure to electrically shocked-conspecific suppressed operant responding in rats [2] and pigeons [3]. More interestingly, experience of exposing electric shock to an observer resulted in the strong suppression of operant responding by distress response by others. These results demonstrate aversive property of distressed conspecific. Preston and de Waal gave a review of research of empathy with animals [4]: mice which observed other mice bitten by biting flies showed more self-burring response when exposed to flies [5], as well as elevated level of corticosterone [6]. Langford demonstrated that the pain response of mice enhances pain response in others [7]. These results show that common experience causes an empathetic response, which results in facilitation of the aversion in the empathizer.

Stress also has an interesting effect on memory, particularly that from an aversive experience as shown by Roozendaal [8]. Miracle gave rats restrain stress for 7 days and examined retention of tone-shock conditioning after extinction of the conditioning [9]. The rats, which received the stress, showed stronger retention or natural recovery 2 days after the extinction when compared with the control rats. Therefore, the rats that experienced stress were more resistant to extinction of fear conditioning than those who did not experience stress. Chronic food deprivation, another type of stress, also caused enhanced retention of passive avoidance in C57/BL mice [10]. Cabib showed that the memory of aversive memory was enhanced by injection of corticosterone dose dependently [11].

One of the interesting emotions in humans is so-called “Schadenfreude”. Distress of others is not aversion but pleasure for observers in the Schadenfreude. Japanese expression of the Schadenfreude is “unhappiness of others is a taste of honey”. The reversed-empathy is mirror image of this phenomenon, that is, “happiness of the others is a taste of acid”. Here we examine empathy based on common experience and reversed empathy based on uneven experience using the memory enhancing effects of stress.

## Methods

### Subjects

80 male C57/BL6 mice were used for the behavioral experiment. 5 mice lived together in one cage. 4 of 5 mice in each cage of a reversed empathy group (described below) were used as cage mates not as subjects of conditioning experiment. Thus, in total 40 mice were used as the cage mate. Other 40 mice including 1 mouse in each cage of the reversed empathy group were used for conditioning. They were 8-weeks after birth when the experiment started. Five mice lived in a cage. Food and water were freely available in the cages. Temperature was kept at 24 degrees Celsius and light-dark cycle was reversed 12L: 12D condition. Another 30 mice (male C57/BL6) were used for measurement of corticosterone. They were 8 weeks at the start of experiment. All animals were treated in accordance with the Guideline of Japanese Society of Animal Psychology and was approved by the Animal Care and Use Committee of Keio University (No08011).

### Effects of empathy and reversed-empathy on aversive memory

#### Apparatus

An experimental chamber (20×20×18 cm) made of transparent acryl was used. The floor was a grid floor with 1.5 cm intervals. Scrambled electric shock was given by a shocker (MED, AND AC 8721). The ceiling had a rectangular hole (10×10 cm) through which the subject was inserted or removed. An artificial marble cube (5×5×4 cm) was used as a step. To restrain animals, a mouse holder for sampling blood (ASONE, To-12B-508-02) was used. The holder (diameter 30 mm, length 100 mm) was made of transparent acryl and the subject could not turn around in it. A white noise (75dB) was broadcasted throughout the experiment.

#### Procedure

**Figure 1:**
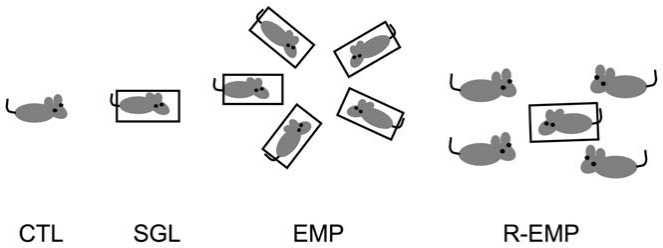
Illustration of four groups. CTL: control group that received no stress, SGL: single stress group that received stress alone, EMP: empathy group that received stress in group with cage mates, and R-EMP: reversed empathy group that received stress with no-stress cage mates.

Handling: All subjects received handling for 10 days each lasting around 5 min. Stress: The subjects were divided into 4 groups of 10 subjects. Single stress group (SGL) received restrain stress for 2 hrs. The subjects were inserted into the holder and a lid was fixed. The holder was placed in a wooden cage with wire-mesh window (30×30×20 cm). Light of the experimental room and white noise were turned on during the 2 Hrs. Then the subjects were removed from the holder and returned to their living cage. The wooded cage was wiped with 70% ethanol and dried. This procedure was repeated for 7 days. Empathy group (EMP) received the same procedure but all five subjects were inserted into the holders and placed in a cage. The holders were placed in the radial heading to the centre so that each animal could see each other. Spatial arrangement of the holders was fixed during the 7 days. Reversed empathy group (R-EMP) received treatment identical to the empathy group except that only one mouse was restrained in the holder with four freely moving cage mates. Control (CTL) group did not receive stress and were not restrained. Figure 1 illustrates the four groups. Passive avoidance training: The subject was gently placed on a step in the experimental chamber. When all four legs touched the grid floor, scrambled electric shock (0.1 mA) was given repeatedly every 1 sec until the mouse climbed up to the step. If the mouse stepped down to the floor within 5 min, the same procedure was repeated. If the mouse stayed on the step for 5 min, the training was finished and extinction procedure began. The step was removed and the mouse was placed on the floor without any electric shock. After 10 min, the mouse was returned to the home cage. Memory testing: One hour after the extinction, the mouse was placed on the step again and time to step down to the floor was measured. The step down was defined as touching the floor with four legs. No electric shock was given. When the animal did not step down to the floor for 10 min, it was placed on the floor for 30 sec. This procedure was repeated three times, 1 hr after the extinction, then 1 day, 3 days and 7 days after the extinction.

### Measurement of corticosterone after stress

#### Apparatus

To restrain animals, a mouse holder for sampling blood (ASONE, To-12B-508-02) was used. The holder was made of transparent acryl and a mouse cannot turn around in it.

#### Procedure

Procedure to give the stress was exactly the same as that in behavioral experiment. Single stress (SGL) group (N = 10) received the stress alone, empathy (EMP) groups (N = 10) received the stress with cage mates, and reversed empathy (R-EMP) group (N = 10) received the stress with non-stressed cage mates. Radioimmunoassay of corticosterone: Mice were scarified by cervical dislocation and 0.1– 0.2 ml blood sample was obtained from the hearts immediately. Then plasma was obtained after centrifugation (14000/min) of the blood samples for 10 min and kept in −80 centigrade. The plasma was sent to SRL Ltd. and chromatographed by Sephadex LH-20, then corticosterone-1, 2–3 H 10000 dpm was added to the extract and corticosterone was counted by a liquid scintillater (LSC5100). See Nabor et al [12] for detailed procedure.

## Results

### Effects of empathy and reversed-empathy on aversive memory

**Figure 2:**
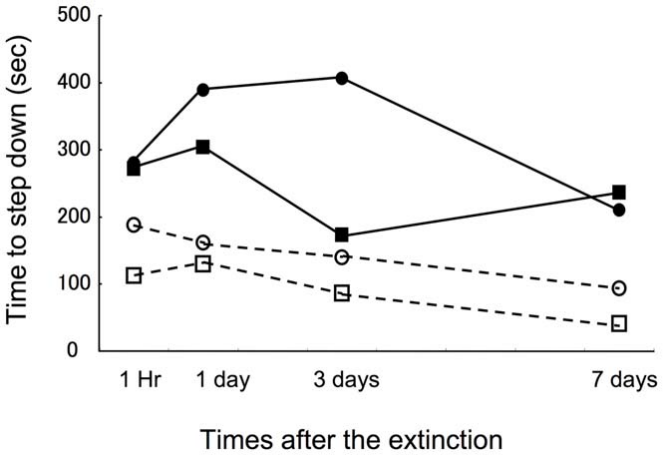
Time to step down to the floor starting from when the subjects received electric shock during conditioning. Each group received 10 min extinction after the conditioning then, received test after 1 hr, 1 day, 3 days and 7 days. CTL: control group that received no stress, SGL: single stress group that received stress alone, EMP: empathy group that received stress in group with cage mates, and R-EMP: reversed empathy group that received stress with no-stress cage mates. N = 10 in each group.

Figure 2 shows mean time compared to step down within each group. Control group (CTL) easily learned to step down on the floor after extinction but the single stress group (SGL) hesitated to step down, particularly on day 1. Interestingly, the empathy group (EMP) did not show such effect suggesting common stress experience reduced the memory enhancing effect of the stress. The largest effect was observed in the reversed empathy group (R-EMP), which clearly showed hesitation to step down 3 days after the extinction. Two factors ANOVA (groups x days) gives main effect of the groups (F (3/144) = 22.52, P<0.001), and days (F (3/144) = 3.78, P<0.05) but no effect of their interaction (F (9/144) = 2.00, P = 0.19). Two factors ANOVA with the SGL and the EMP groups reveals significant effect of the groups (F (1/72) = 9.50, P<0.01) but no effect of the days or interaction (F (3/72) = 1.57, p = 0.20 and 0,65, p = 0.58, respectively), suggesting sharing stress in a group reduced the effect of the stress. ANOVA with the SGL and R-EMP groups reveals significant effect of the groups ((F (1/72) = 4.22 P<0.05) but no effects of the days and interaction (F (3/72) = 1.90 and 2.47, p = 0.14 and 0.07 respectively), suggesting experience of stress with non-stressed cage mates enhanced effect of the stress. ANOVA with the EMP and R-EMP groups reveals significant effect of the groups ((F (1/72) = 32.85 P<0.0001) and days (F (3/72) = 3.50, P<0.05) but no effects of the interaction (F (3/72) = 1.88, P = 0.14). These analysis show common experience of stress reduces the memory-enhancing effects of stress. Stress experience with non-stressed conspecific, however, does enhance the stress effects on memory.

### Measurement of corticosterone after stress

**Figure 3:**
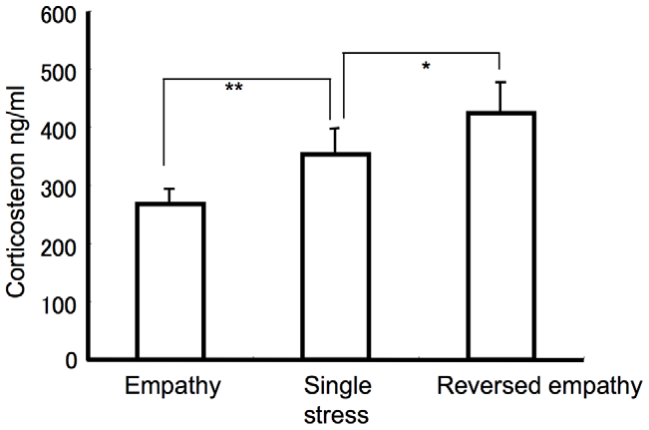
Corticosterone levels after 1 hour restrained stress. SGL (single stress group) received the stress alone, EMP (empathy group) received the stress with cages mates and R-EMP (reversed empathy group) received stress with non-restrained cage mates. N = 10 in each group. **P<0.05, *P<0.10.

Figure 3 showed the corticosterone level in the three groups. One factor ANOVA reveals significant effect of the groups (F (2/14) = 15.59, P<0.001). A t-test shows a significant difference between the single stress and empathy groups (t(8) = 3.46, P<0.01) and between the single and reversed empathy groups (t(8) = 2.15, P<0.05). Thus, the social situation during stress induction did modify corticosterone level. Sharing stress reduced the effect of the stress and stress with non-stressed cage mates enhanced the stress.

## Discussion

Behavioral experiment shows 1) memory enhancing effects on aversive conditioning, 2) common experience of stress with the cage mates reduces the enhancing effects, but 3) stress with non-stressed cage mates facilitated the enhancing effects of stress. That is, the empathy decreased the stress but the reversed empathy increased the stress. Thus, restrain experience caused different amount of stress depending on social situations even though the restrain time was the same. I found social facilitation of reinforcing effect of methamphetamine after conditioning together with cage mates but drug-free cage mates did not have such social facilitation effect [13]. Thus, the social facilitation depends on state of social partners. Other researches also suggest that empathetic response is affected by relative status of observer and demonstrator. Mice injected with higher concentration of formalin (strong pain) reduced their pain response by observing mates injected with lower concentration (weak pain), while those with weak pain increased their pain response by observing mate with strong pain [7]. These theories support the present results. The contradictory effects between the empathy group and the reversed empathy group may suggest precursor of “sense of fairness”. Several studies suggest human social intelligence has a biased sense of fairness, hence humans want to punish others unfair behavior even if they have to pay for the punishment [14]. Non-human animals study indicates some kind of sense of fairness in primates. Capuchin monkeys refused cucumbers when others obtained grapes instead of cucumbers [15]. Thus, relative status with the others determines the monkeys' behavior. Of course we have to carefully examine details of factors that enhance the stress effect. This supports the present data, as the R-EMP groups demonstrated this idea of fairness in their enhanced effects of stress, whereas they were decreased in the EMP rats due to the social equality.

Measurement of corticosterone presents levels of the corticosterone depends on social context. The results correlate with those from the behavioral experiment and suggest memory modification through levels of corticosterone. The stress in the behavioral experiment was acute one rather than chronic one, but memory modification by stress in can be explained in terms of level of the corticosterone. Memory enhancement by elevated corticosterone was confirmed by injection of corticosterone in rats [11]. The rats injected with corticosterone showed enhanced memory of passive avoidance depending on the dosage. Therefore, the empathy reduced the corticosterone level and resulted in a lack of memory enhancement, whereas the reversed empathy increased the corticosterone level and resulted in memory enhancement.
